# Now, I think doctors can be heroes … Medical student’s attitudes towards the COVID-19 pandemic’s impact on key aspects of medical education and how the image of the medical profession has changed due to the COVID-19 pandemic

**DOI:** 10.1007/s10354-021-00891-1

**Published:** 2021-10-29

**Authors:** Verena Steiner-Hofbauer, Julia S. Grundnig, Viktoria Drexler, Anita Holzinger

**Affiliations:** Teaching Center, Medical University of Vienna, Spitalgasse 23, 1090 Vienna, Austria

**Keywords:** Medical Students, Corona Virus, Distance learning, Qualitative Data, Occupational Image, Medizinstudierende, Corona Virus, Fernlehre, Qualitative Daten, Berufsbild

## Abstract

**Background:**

The COVID-19 (coronavirus disease 2019) pandemic hit the world in early 2020 and influenced medical education worldwide. Distance learning, risk of infection and patient care, telehealth literacy, medical ethics and research in medical education are key factors of medical education challenged by the pandemic. Additionally, the following question arises: “What do medical students think about their future profession in the face of this crisis?”

**Methods:**

A cross-sectional survey was conducted among all undergraduate medical students of the Medical University of Vienna. 872 students answered the self-developed questionnaire. Qualitative and quantitative data analyses were conducted.

**Results:**

The data show that the COVID-19 pandemic has raised awareness for the key aspects. In all areas of interest, students’ feeling of petaredness is limited. Limitations in practical training and distance learning as well as social isolation concern a majority of students. Neutral, positive, and negative themes emerged in qualitative data analysis. Only 8% of the students of the first 3 years of study versus 13.4% of the students in higher years commented negatively. 18.7% of male vs. only 12.5% of all female students’ comments were positive. A large proportion of positive comments were dedicated to the relevance and deeper meaning of the medical profession. Infection risk and the demanding nature of the medical profession were predominant in negative comments.

**Conclusion:**

The COVID-19 crisis has turned the spotlight on several aspects of medical education in need of reform. In addition, the occupational image of the medical profession seems to shift under the weight of this pandemic.

## Abstract (de)

**Hintergrund:**

Die COVID-19-Pandemie (Corona Virus Disease 2019) traf die Welt Anfang des Jahres 2020 und hatte auch Einfluss auf die medizinische Ausbildung weltweit. Fernlehre, Infektionsrisiko und Patientenversorgung, Kompetenz in Bezug auf Telemedizin, medizinische Ethik und Forschung sind wesentliche Faktoren der medizinischen Ausbildung, welche durch die Pandemie vor Herausforderungen gestellt wurden. Darüber hinaus ergibt sich die folgende Frage: Was denken Medizinstudierende angesichts dieser Krise über ihren zukünftigen Beruf?

**Methodik:**

Unter allen Medizinstudierenden der Medizinischen Universität Wien wurde eine Querschnittstudie durchgeführt. Den selbstentwickelten Fragebogen beantworteten 872 Studierende. Es wurden qualitative und quantitative Datenanalysen vorgenommen.

**Ergebnisse:**

Die COVID-19-Pandemie hat das Bewusstsein für die untersuchten Faktoren geschärft. In allen untersuchten Schlüsselbereichen ist das Gefühl der Vorbereitung der Studierenden mäßig. Einschränkungen in der praktischen Ausbildung, durch Fernlehre sowie soziale Isolation bereiten der Mehrheit der Studierenden Sorgen. In der qualitativen Datenanalyse fanden sich neutrale, positive und negative Themen. Nur 8% der Studierenden aus den ersten 3 Studienjahren gegenüber 13,4% der Studierenden aus den höheren Jahren gaben negative Kommentare ab. Positiv waren 18,7% der Kommentare der Studenten vs. nur 12,5% derer der Studentinnen. Ein großer Teil der positiven Kommentare befasste sich mit der Relevanz und der tieferen Bedeutung des Arzt‑/Ärztinnenberufs. Infektionsrisiko und die anspruchsvolle Natur des Berufs herrschten in den negativen Kommentaren vor.

**Schlussfolgerung:**

Die COVID-19-Krise hat den Fokus auf verschiedene, reformbedürftige Aspekte der medizinischen Ausbildung gerichtet. Darüber hinaus scheint sich das Berufsbild des Arzt‑/Ärztinnenberufs durch die Pandemie zu verändern.

## Introduction

The COVID-19 (coronavirus disease 2019) pandemic hit the world in early 2020. For most people in developed countries, it is the first health crisis ever experienced. It influenced and still influences not only our private life due to social distancing, lockdowns, and curfews, but it also impacts medical education worldwide [1]. Distance learning, seminars with attendance, and clinical placements under the risk of infection became immediate problems to solve for universities, but also subsequent challenges are interwoven.

In March 2020, Austrian universities were forced to switch rapidly to online teaching and assessment [2, 3]. Even without the special challenge of distance learning, medical students are often exposed to performance pressure and suffer from impaired mental health [4, 5]. Guse et al. showed that students feel burdened by the effects of the pandemic, that their motivation is decreased, and that many students are worried that the situation will impact their education negatively [6]. “Worried” was the most often mentioned emotion towards medical education in a study of Polujanski et al. [7]. For students already involved in patient care, the situation could be especially troublesome, as studies show that health care workers exposed to COVID-19 show a high prevalence of psychological problems [8, 9]. Additional challenges for the future medical workforce are personal safety during patient care, telehealth literacy, ethical aspects in pandemic planning like triage or elective surgery, and the importance and impact of medical research for the development of test and treatment methods or vaccines [1, 3, 10–12].

Before the pandemic, telehealth/telemedicine was a neglected topic and the increased use during the pandemic was a necessity, not a choice [13]. Medical education must catch up and include empathy in telehealthcare teaching “webside manner” as well as bedside manner [14].

However, not all doctor–patient encounters are manageable via telemedicine. Treating patients bears a special risk of contracting the disease. Nevertheless, medical students rely on clinical practical training to finish their education, as well as the health care system relies on the work medical students contribute to the system [15].

Even before the pandemic, the Liaison Committee on Medical Education stated that every medical education program should include instructions in medical ethics and humanities [16]. The COVID-19 pandemic was a cruel reminder of the impact of ethical decisions on patients’ health and life—decisions like who gets a ventilator or an intensive care bed when not everyone can, or which surgery is not urgent enough and can be postponed despite a clear medical need, and many more [11, 17]. Additionally, there is a moral obligation to learn and conduct research, and to work on vaccines, drugs, and new treatment methods [17].

This leads to another field of medicine exposed to keen interest and harsh critique due to this pandemic [18]. Literature suggests that research is often underrepresented in the curricula; therefore, students are ill prepared in epidemiology, statistics, and scientific investigation [18–20]. Kyaw et al. also conclude that a supportive and positive environment is needed to improve skills and knowledge and thereby encourage students to conduct research [21].

In addition to these topics, other questions arise: How has this pandemic affected the image of being a doctor for the students? Has the image of the medical profession changed?

These are key aspects for present and future medical education, but research on the attitudes of medical students towards these topics is lacking.

This study aims to explore:

- Agreement/disagreement with certain statements about research, ethics, telemedicine, and safety during patient care in the face of the COVID-19 pandemic.
- Students’ subjective feeling of preparedness in the abovementioned key areas.
- Students’ attitudes towards distance learning and social distancing.
- How the COVID-19 crisis has changed the occupational image of the medical profession.

## Methods

To answer these questions, a cross-sectional survey was conducted among all undergraduate medical students of the Medical University of Vienna. The data were collected via the internal online evaluation tool of the Medical University of Vienna. The questionnaire was online between December 2, 2020, and January 31, 2021; all students received an invitation via their student email account.

The internal data protection commission as well as the internal research clearing board of the Medical University of Vienna approved this study.

### Sample

A total of 872 students answered the questionnaire, this represents around 15% of all students of the Medical University of Vienna. 485 female (56.3%), 365 male (42.4%), and 4 (0.5%) nonbinary students participated, 7 students (0.8%) choose “no declaration” as gender option. On the date of answering the questionnaire, 2.1% of the participants were younger than 19 years, 33.2% were between 19 and 21 years, 37% from 22 to 24 years, 15.3% between 25 and 27, 6% between 28 and 30, 2.7% between 31 and 33, and 3.8% older than 33 years. Of the participating students, 264 were in their first year of study, 160 in the second, 142 in the third, 126 in the fourth, 85 in the fifth, 59 in the sixth, and 18 above the sixth year of study. The majority of participants were medical students (89.9%), 10.1% students of dentisty participated.

### Questionnaire

Based on the literature, we developed a questionnaire that included the following topics: research, ethics, safety, patient care, telemedicine, and distance learning. It contained closed questions using a 5-point Likert scale, ranging from 0 = “I totally do not agree” to 4 = “I totally agree,” and an open-answer section for the question about the occupational image. The questions concern general attitudinal aspects as well as specific aspects of education at the Medical University of Vienna.

### Statistical analysis

Descriptive statistics are stated as the sum of agreement. Therefore, the answers “I totally agree (4)” and “I agree (3)” were summed up. Undecided answers (2), “I do not agree (1),” and “I totally do not agree (0)” were therefore excluded from the description. To determine differences between “beginners” and “advanced” students, the participants were split into two groups: the beginner group ranging from the first to the third year of study, the advanced group ranging from the fourth to the seventh year of study. This separation is based on the standard length of the medical course in Vienna, which is 6 years.

For missing data, listwise exclusion was chosen, as no variable showed more than 10% missing values and 75% of all cases showed no missing values. For statistical analysis, SPSS 24.0 (IBM, Armonk, NY, USA) was used [22]. T‑tests were used to calculate the significance of mean differences.

### Qualitative data analysis

Qualitative data were analyzed following the qualitative content analysis of Mayring. The software MAXQDA (VERBI Software. Consult. Sozialforschung GmbH, Berlin, Germany) was used for analysis [23]. Different coding of statements was resolved by discussion and agreement. Differences between beginners and advanced students were determined.

## Results

### Research

The answers regarding changes in awareness of research and its dynamic due to the COVID-19 pandemic showed that 50.6% of all participants became aware of the fact that research findings are dynamic and do not represent absolute truths. 52.7% confirmed that they became aware that research findings can lose their validity within a short time. 55% reported that they became aware of how long the development of drugs and vaccines takes through the COVID-19 pandemic.

Only 22% of all students stated that the COVID-19 pandemic had raised awareness for the significance of medical research and only 16.9% would choose medical research as an occupational field because of the pandemic as well as only 10.8% of the participants think that people of other fields of expertise, like biologists or pharmacists, should mainly work in medical research.

Table 1 shows the students’ evaluation of research at the Medical University of Vienna.

**Table 1:** Agreement towards questions regarding research

Question	Agreement (in %)
Feel prepared for a career in research	37.2
Enough time for research and its significance in the curriculum	36.3
Research literacy is essential for clinical work	72.6
*Personal research experience*	
Did participate in a research project at the university	20.3
First authored/co-authored a research paper	6.8
Presented research results at a conference	5.5

**Fig. 1:**
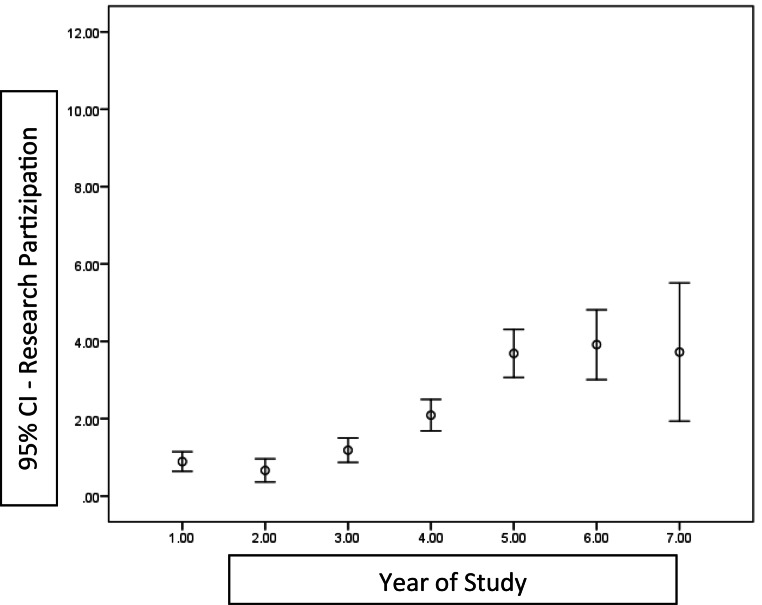
Research participation per year of study

Fig. 1 shows research participation of the students over the course of the study as mean sum of the answers to the questions concerning “Personal research experience.”

### Ethics

Responses regarding whether awareness of research and its dynamics have changed due to the COVID-19 pandemic showed that 32% of students became aware that medicine and ethics are intertwined. 53.5% of all participants became aware that doctors have to decide who will get a lifesaving treatment and who will not if the capacities are limited. 61.1% became aware that doctors have to decide which treatments and surgeries have to be postponed, even if the suffering of patients will be prolonged, and 49.6% became aware of the fact that triage and postponing treatments could lead to the death of certain patients. That doctors influence political decisions in general came into awareness of 51.3%. Only 2.3% of all participants thought that mainly people of other fields of expertise, like philosophers or jurists, should render ethical decisions for medicine.

Table 2 shows the students’ evaluation of ethical aspects at the Medical University of Vienna.

**Table 2:** Agreement towards questions regarding ethics

Question	Agreement (in %)
Feel prepared for ethical challenges	51.9
Enough time for ethics and its significance in the curriculum	47.6

### Telehealth/telemedicine

The answers regarding changes in awareness of telemedicine and its significance due to the COVID-19 pandemic showed that 53% of all participants became aware of the significance of telehealth. Only 26.1% see the future of medicine in telehealth. 23.6% of the participants recognize telehealth as a solution for challenges to security of supply in general medicine and 29.3% as a solution for general medicine in rural areas. Only 12% of the students think that personal contact between doctor and patient is often unnecessary, but 79.8% think that in specific areas, telemedicine is useful and should be implemented permanently.

Table 3 shows the students’ evaluation of telehealth aspects at the Medical University of Vienna.

**Table 3:** Agreement towards questions regarding telehealth

Question	Agreement (in %)
Feel prepared for challenges of telehealth	9.2
Enough time for telehealth and its significance in the curriculum	6.9
Necessity of integrating telehealth throughout the curriculum	39.2

### Preparedness

Subjective feeling of preparedness was defined as the mean sum of the answers to the questions “Feel prepared for challenges” and “Enough time is spent in the curriculum.”

Table 4 shows the feeling of preparedness of the students during the first half of their studies (years 1–3) and the second half of their studies (years 4–7).

**Table 4:** Means of feeling of preparedness

Field	Mean year 1–3 (SD)	Mean year 4–7 (SD)	*p*-value
Research	4.75 (1.75)	3.73 (2.02)	0.000*
Ethics	5.08 (1.84)	4.43 (2.19)	0.000*
Telehealth	3.13 (1.68)	1.55 (1.75)	0.000*

*SD* standard deviation (in parentheses)

*Significant difference between beginners and advanced students, *p* < 0.05

### Infection risk

The answers regarding changes in awareness of infection risk in the medical profession due to the COVID-19 pandemic showed that 37.8% of the participants became aware of the health risks related to a career in medicine, but only 4.7% reported that the image of the medical profession had changed to the negative due to this fact. Only 3.6% agreed with the statement that they would only choose an occupational field without a high infection risk.

Table 5 shows the students’ evaluation of infection risk aspects at the Medical University of Vienna.

**Table 5:** Agreement towards questions regarding infection risk

Question	Agreement (in %)
Feel prepared for safety aspects regarding infection risk	52.8
Only specially trained doctors should work with infectious patients	44.5
*Personal safety in COVID-19 context*	
Maintaining high standards for infection prevention (hand sanitation, distance, mask)	95.7
Following the rules of the government	81.3
Medical students bear greater responsibility than the general public	58.2
Trying to inform and educate others regarding infection prevention	83.7

### Patient contact and infection risk

Regarding direct patient contact during the practical training, 58.8% of the participating students report that their patient contact was severely limited, 46.5% of the students think that the practical training they receive is insufficient for their future in the workforce. Only 9% experienced no changes in practical training. Special training for the safe handling of infectious patients was received by 23.8% of the participants. Only 8.9% state that they would prefer to have no patient contact until vaccination is available for them. 19.5% think that infection risk is a common topic in medicine, and, therefore, COVID-19 makes no difference to them. A majority of students, 56.4%, state that they feel an intensified need to use their knowledge and skills to help overcome this crisis.

### Distance learning

Table 6 shows the students’ evaluation of distance at the Medical University of Vienna.

**Table 6:** Agreement towards questions regarding distance learning

Question	Agreement (in %)
Distance learning offers should remain after the pandemic	70
All lectures can take place in the same quality online as in person	56.6
For practical skills training, distance learning represents no alternative to in-person training	89.6
The quality of my education is suffering under the safety measures	69.9
Social aspects and personal contact to fellow students and university staff is irreplaceable for me	84.7
The social isolation due to distance learning burdens me	61.3

### Subjective occupational image

During the qualitative data analysis, neutral, positive, and negative comments emerged, as well as comments indicating that no changes had occurred or that several “current” aspects were already known. Table 7 shows the comments separated into neutral comments including the subsets responsibility, health/system/politics, and professions; positive comments including the subsets relevant/meaningful profession, appreciation/acknowledgment, crisis-proof profession, helping, and assured in career choice; negative comments including the subsets acknowledgment, responsibility, infection risk, demanding profession, negative role models; and “No” changes comments including the subsets “no, risks well known” and “no, responsibilities well known.” In several themes, group differences emerged.

**Table 7:** Emerged themes and subsets (denomination), description of the theme, examples

Code	Description	Example
*Neutral (56)*	Neither positive nor negative comments	It made me aware that you can’t always know everything and of how important it is that doctors and scientists work together. We are only human.
Responsibility (28)	Comments regarding the great responsibility doctors bear	You have to bear great responsibility—more than I thought. I am more aware of the great responsibility than ever.
Health/system/politics (17)	Comments about greater contexts of health in general, the health system, or the connection to politics	I was not aware of how close medicine and politics are intertwined during a pandemic. I became aware of how important a good health system is. It made me aware of how important health is and how everything stops working if it is not ensured.
Professions (12)	Comments about different specialties in medicine	I want to be an infectiologist. My occupational picture was broadened, I learned of new possibilities within the spectrum of medical professions.
*Positive (85)*	Positive comments	I had the classic image of the medical profession: normal job, good money, and respect from society, but not world-shaking. Now I think that doctors can be real heroes and that there is more to this job than meets the eye.
Relevant/meaningful profession (47)	Comments about the deeper meaning and relevance of the medical profession	It underlines the importance of medicine and the people who dedicate their lives to it. It improved. The importance of it was proved.
Appreciation/acknowledgement (20)	Comments about the increased appreciation and acknowledgement of the medical profession	I have even more respect for the incredible achievements of health care workers. I have the feeling that a little respect came back—before I had the feeling that doctors are not much respected and acknowledged.
Crisis-proof profession (7)	Comments underlining the profession’s safety against crises	Yes, it showed me that this job is safe during crises and that doctors are always needed. The pandemic has underlined that jobs in the health system are safe jobs.
Helping (6)	Comments about the chance to help others	I want to help people more than ever, and I will try everything to become a good doctor. The picture of the job has changed positively. You can help so many people.
Assured in career choice (13)	Comments underlining the fact that students felt assured about their career choice	I am assured in my choice to become a doctor. For me personally, the pandemic intensified my desire to become a doctor. COVID-19 was a reason for me applying at the university.
*Negative (58)*	Negative comments	It underlined the helplessness of doctors when effective treatments and drugs are lacking.
Acknowledgement (10)	Comments about the decreased or lacking appreciation and acknowledgement of the medical profession	I became aware of how little respect the general population has for doctors and nurses. The reputation of this job is not what it was 10 years ago. I am more aware of the imbalance in our society than ever, that doctors do not earn enough compared to other professions.
Responsibility (8)	Comments about the great responsibility of the medical profession	It is very burdensome to think about the great responsibility that you have to bear in the future. It showed me which hard choices doctors have to make, and how they suffer under this burden—especially during the pandemic.
Infection risk (17)	Comments underlining the burden of the personal infection risk	I thought that the job was not that dangerous. I became aware of how this job jeopardizes your own and your family’s health.
Demanding profession (22)	Comments about the demanding and overextending nature of the medical profession	It showed me how demanding this job can be—especially because of staff shortages. It showed me how demanding this job is, and it pushes people to their limits to help others.
Negative role models (16)	Comments about the negative role models that emerged during the COVID-19 crisis	For me, the picture changed towards the negative. I recognized that doctors closed their practices and let their patients down because they were scared. Sadly, so many doctors deny scientific facts and distrust science.
*No (147)*	Comments indicating that no changes occurred	No. Not really. Barely.
No, risks well known (12)	Comments underlining that the personal health risks of medical profession were well known	No, I knew the risks in advance. You can protect yourself and help people who cannot. No, infection risk was always relevant.
No, responsibilities well known (8)	Comments underlining that the great responsibilities of the medical profession were well known	Doctors bore great responsibilities before. No, I knew that doctors bear great responsibility

Denominations of the subsets do not sum up because of the possibility of multiple assignment

### Gender differences

The analysis of gender differences showed that 18.7% of all male students’ comments were positive, whereas only 12.5% of all female students’ comments were considered positive. All other sets and subsets showed differences only < 5%. Including all subsets, the difference is 22% vs. 30.1%. Male students commented more often in the subsets “appreciation and acknowledgment” and “relevant/meaningful profession,” whereas female students commented more often in the subsets “crisis-proof profession” and “assured in the career choice.”

### Differences in progress/year of study

The analysis of differences between students in the first, second, and third year of the study compared with students in the fourth or higher years of the study showed that 8% of all comments of the students in the first 3 years of study were considered negative versus 13.4% of all comments of students of higher years. Respectively, 16.6% vs. 12% were positive. All other sets and subsets showed differences only < 4%. Students of the first 3 years commented especially in the category “relevant and meaningful profession.”

## Discussion

This study aimed to shed light on the COVID-19 pandemic’s influence on key aspects of medical education, i.e., research, ethics, telemedicine, infection risk, and patient care, as well as distance learning. The data showed that the COVID-19 pandemic has raised awareness for the key aspects, which were content of the questionnaire. In all key aspects, the feeling of preparedness of students is limited. Constraints in practical training, distance learning, and social isolation concern a majority of students. Most students are strongly aware of personal security measures and, as medical students, feel a special responsibility to be role models and inform the public about safety measures. Neutral, positive, and negative themes emerged in the qualitative data analysis. For male and younger students, the changes seem to be more positive. A large proportion of positive comments was dedicated to the relevance and deeper meaning of the medical profession. Infection risk and the demanding nature of the medical profession were predominant in negative comments.

A lifelong commitment to reflective learning, the ability to evaluate information and its sources critically, and contribute to the creation, dissemination, application, and translation of new medical knowledge is crucial for all physicians [24]. Even though 70% of all students think research is essential for clinical practice, only 37% feel prepared for a research career or agree that the university invests enough time on research and its significance. This lack of focus on research may result in low numbers of research engagements and publications. Top US Universities Stanford and Duke have publication rates of up to 75% during mandatory research education [25]. In our study, only around 7% first- or co-authored a paper, and only 20% engaged in a research project at the university. Most research activities start towards the end of the study, during the fourth or fifth year. Chang and Ramnanan found that early research experiences may be a motivating factor for research engagement; simultaneously with the duration of research experiences, first authorships increase, and students’ perceptions of research experiences are enhanced [26]. For universities, this is a signal to encourage and promote students’ early involvement in research and build an environment that offers suitable opportunities in which to participate. Only a minority of students think that experts in other fields should primarily conduct medical research; therefore, medical education has to prepare students for a research career properly. Interestingly, students of the first 3 years feel better prepared for research as well as for ethical and telehealth challenges than students towards the end of their studies. Reasons for this may include that the whole extent of subject matter is unclear at the beginning of the course or that students at the end of their studies—facing real responsibility in the near future—tend toward a more modest assessment of their abilities.

In professional ethics, two topics of this study come close: Personal infection risk and professional decision-making in times of limited medical care options. McChullough et al. took up this topic and developed a framework for teaching professional formation between the poles “taking reasonable risks” and “self-sacrificing” [27]. In our study, only about half of the participants felt prepared for the ethical challenges. Many participants only became aware of the extensive ethical aspects of medicine due to the pandemic. The great responsibility going hand in hand with the medical profession was one of the major topics in the qualitative data—often with a negative connotation. Therefore, it seems especially important to support and prepare students with expertise in this field. Up until the COVID-19 pandemic, rationing and scarcity were uncommon terms in medical care in Western countries and also in Austria. The pandemic has raised several questions about these topics [28]. Han et al. identified allocation of scarce resources as the dilemma arising from the pandemic and refer to guidelines to “lift the moral accountability of each decision out of the hands of the few at the bedside and distribute it among a collective conscience” [29]. They also recommend introducing trainees to an active conversation about basic ethical principles like principles of maximization of benefit, equal treatment of all patients, and priority to the sickest. DeFoor and colleagues found that there is a strong and often unmet need and interest in formal ethics education [30]. Accordingly, only 2.3% of our participants would prefer other experts to render ethical decisions for medicine, even if only half of all students feel prepared for this challenge themselves.

However, personal risk is also a topic highlighted by this pandemic; this is an ethical problem, too. The code of ethics of the American Medical Association states clearly that taking reasonable risks is an ethical obligation for medical professionals—this might be the heroic part of this profession—but taking unreasonable risks is not heroic but reckless [16]. In our study, about 40% of the participants had become aware of this topic due to the pandemic. Only half of all participants feel prepared for infection risks and, accordingly, almost as many think that only specially trained doctors should care for infectious patients. Even though infection risk was an often negatively connoted topic in qualitative data, the participants found clear words for doctors who hide from their ethical obligation and abandon their patients or turn away from their roots in science. This leads to another ethical obligation held by the medical profession: identifying means to prevent pandemics and educate the public about them [31]. The participating students widely agreed that they try to inform and educate others about infection prevention, and more than half of them felt they bear a special responsibility to do so as medical students. Most of them were also ready to follow governmental rules to prevent the virus from spreading. Accordingly, Komasawa et al. found in a sample of Japanese medical students that they exercised self-control regarding their lifestyle [32].

An important way of reducing infection risk for medical professionals is switching from in-person treatment to telehealth consultations. Muntz et al. outline the possibility that limited clinical internships could be replaced by telehealth activities [33]. Both the health care system as well as the students would profit. In our study, only a minority of students feel prepared for the challenges of telehealth. For most of them, the personal contact between patient and doctor is irreplaceable. Despite this fact, almost 40% see a need for implementing telehealth throughout the curriculum. Dawidzuik and Grandhewar underlined the importance of digital health literacy, especially in handling mass information from the internet, and the challenges of communicating complex information to patients [34]. These findings are a clear wake-up call for medical educators.

Another way of keeping students safe is distance learning. The students participating in this study see positive aspects of distance learning and hope that several distance-learning offers will remain after the pandemic. Nevertheless, the social isolation coming with distance learning burdens more than 60% of the participants. Accordingly, Silva et al. found that students’ quality of life was severely impaired by distance learning. They recommend interaction with professors as a key coping tool [35]. The students in this study also feel that contact with university staff and fellow students is irreplaceable. Almost 60% of all students report severely limited patient contact, and 46.5% of all students think that their practical skills training is insufficient for future practice. Bellini et al. propose that presumably, the whole educational offer from the academic institutions will learn from this pandemic and evolve to a changed model, where new and traditional formats will work side by side [36].

Despite the different challenges and problems the COVID-19 pandemic causes, many students expressed that this situation intensified their wish to become doctors and help to overcome this or future crises. In this study, the positive statements outweigh the negative ones, too. Especially male students provided more positive statements, particularly in the subsets “relevant/meaningful profession” and “acknowledgment.” The medical profession is culturally and historically linked to masculinity and male-attributed behavior is still seen as prototypical for physicians [37, 38]. This may be a reason why male students more often claim the relevance and meaningfulness of this profession for themselves than female students do. Female students’ comments highlighted the crisis-proof nature of the medical profession. This underpins the finding of Piasna and Plangnol, that job security and prospective parenthood are closely intertwined only for women [39].

Qualitative analysis revealed that the “beginners” group, compared to the advanced students’ group, was more likely to make positive comments. An overly positive attitude towards the demanding nature of the medical profession may be challenged by the approaching reality of demanding and potentially risky working conditions.

However, Komasawa et al. found that Japanese medical students were passionate about becoming doctors, too [32]. Therefore, the world seems full of passionate young people; for some of them, the deeper meaning of the medical profession has only become clear due to this crisis. As one participant answered:

I had the classic image of the medical profession: normal job, good money, and respect from the society, but not world-shaking. Now I think that doctors can be real heroes and that there is more to this job than meets the eye.

## Limitations

Data were obtained at only one university. Self-selection of participants—through voluntary participation—can always lead to systematically skewed data. Conclusions beyond the surveyed population should be drawn with caution.

During the data-collection phase, Dec 20/Jan 21, the EMA authorized a vaccine. The question formulated with “if a vaccine were to be authorized” was therefore outdated in the middle of the survey. An evaluation leads the authors to the conclusion that the sense and meaning of the question was not impaired by this fact.
